# Use of Miconazole Cream As Adjunctive Therapy to Treat Isolated Sinus Mucormycosis: A Case Report and Literature Review

**DOI:** 10.7759/cureus.69241

**Published:** 2024-09-12

**Authors:** Hussain J Aljubran, Nada A AlBahrani

**Affiliations:** 1 College of Medicine, Imam Abdulrahman Bin Faisal University, Dammam, SAU; 2 Department of Otolaryngology, King Fahd University Hospital, Al Khobar, SAU

**Keywords:** black fungus, fungal rhinosinusitis, fungal sinusitis, miconazole, mucormycosis

## Abstract

Black fungus, also known as mucormycosis, is one of the most serious infections affecting immunocompromised individuals. Invasive fungal sinusitis due to mucormycosis is quite rare globally. Hence, this article presents a case report of invasive fungal sinusitis in a 53-year-old diabetic female who presented to the emergency department with a severe case of diabetic ketoacidosis secondary to acute sinusitis, which was confirmed by histopathology to be mucormycosis. An extensive surgical debridement and liposomal amphotericin B were the mainstay of treatment. The treatment of mucormycosis consists of treating the underlying disease, antifungal therapy, and surgical debridement. Also, previous studies have discussed the use of medical therapy alone, surgical therapy alone, and combination therapy. It was found that the combination of medical and surgical therapy was the most effective method in treating this condition. However, the high mortality rate of this disease indicates the need for a possible adjuvant therapy which could increase the survival rate. Therefore, recent studies have proposed new adjuvant modalities, such as hyperbaric oxygen therapy and local treatment with amphotericin B. In this study, we propose a new adjuvant therapy using local miconazole cream which showed a good prognosis with the combination of oral amphotericin B and surgical debridement. This highlights the necessity for extensive future clinical trials to validate its effectiveness in treating isolated sinus mucormycosis.

## Introduction

Black fungus, also referred to as zygomycosis and phycomycosis, was initially described by Paltauf in 1885 [1]. It was later best characterized by Baker in 1957 as mucormycosis, which is a potentially life-threatening invasive fungal infection [2]. The paranasal sinuses are usually the primary sites affected by mucormycosis, and then they progressively spread to neighboring structures. It is considered a rare condition with a global incidence rate of 0.005 to 1.7 cases per 1,000,000 people annually [3]. This fungus has been linked to the mold fungi of the *Rhizopus*, *Mucor*, *Rhizomucor*, *Cunninghamella*, and *Absidia* genera of the *Mucorales* order [4,5]. Nearly 60% of mucormycosis cases are caused by the most common type of fungus, *Rhizopus oryzae *[6]. Indeed, diabetes mellitus (DM) is the leading global risk factor associated with mucormycosis, with an overall mortality of 46%, although hematological malignancies and organ transplants can also play a role [7]. However, the mortality rate could increase to as high as 50% to 80% in the presence of intracranial or orbital involvement [8]. As mucormycosis is hard to diagnose, it is usually related with poor prognosis. The aim of this case report is to describe a unique case of a diabetic patient with mucormycosis in an academic institution to highlight the significance of timely diagnosis and treatment of this condition. Additionally, this case is reported in line with the Surgical Case Report (SCARE) guidelines [9].

## Case presentation

A 53-year-old female with a 20-year history of type 2 DM and on metformin presented to the emergency department with a three-day history of sore throat, decreased oral intake, and generalized fatigue. The patient was then admitted as a case of diabetic ketoacidosis (DKA) secondary to acute sinusitis and oral thrush and was started on fluconazole. Two days preceding admission, she was noticed to have a fever (38.7 degrees) and she complained of pain on the left side of her face that radiated to her ears, for which she was started on clindamycin. There was no history of a recent infection, and her family history was unremarkable.

Table 1 shows the initial laboratory tests drawn in the emergency department, which were significant for leukocytosis (white blood cells: 20.6 k/µL), thrombocytosis (platelet: 499 k/µL and mean platelet volume: 6.9 fL), slightly reduced mean corpuscular hemoglobin (26.7 pg), slightly reduced mean corpuscular hemoglobin concentration (31 g/dL), elevated hemoglobin A1c (17.4%), elevated inflammatory markers (C-reactive protein: 14.6 mg/dL and erythrocyte sedimentation rate: 108 mm/hr), and elevated procalcitonin (0.48 ng/mL).

**Table 1:** Initial laboratory findings during the emergency department visit

Complete blood count	Reference range	Patient values
White blood cell (WBC)	4-11 k/µL	20.6
Hemoglobin	12-16 g/dL	12.9
Red blood cell (RBC) count	4.2-5.5 Mil/µL	4.83
Hematocrit	37-47%	41.5
Mean corpuscular volume (MCV)	80-94 fL	86
Mean corpuscular hemoglobin (MCH)	27-32 pg	26.7
Mean corpuscular hemoglobin concentration (MCHC)	32-36 g/dL	31
Red cell distribution width (RDW)	11.5-14.5%	12.4
Platelet	140-450 k/µL	499
Mean platelet volume (MPV)	7.2-11.1 fL	6.9
Point-of-care testing (POCT) glucose	70-140 mg/dL	291
Hemoglobin A1c (HbA1c)	4-6%	17.4
C-reactive protein (CRP)	0.1-0.5 mg/dL	14.6
Erythrocyte sedimentation rate (ESR)	0-20 mm/hr	108
Procalcitonin	Less than 0.1 ng/mL	0.48

**Figure 1:**
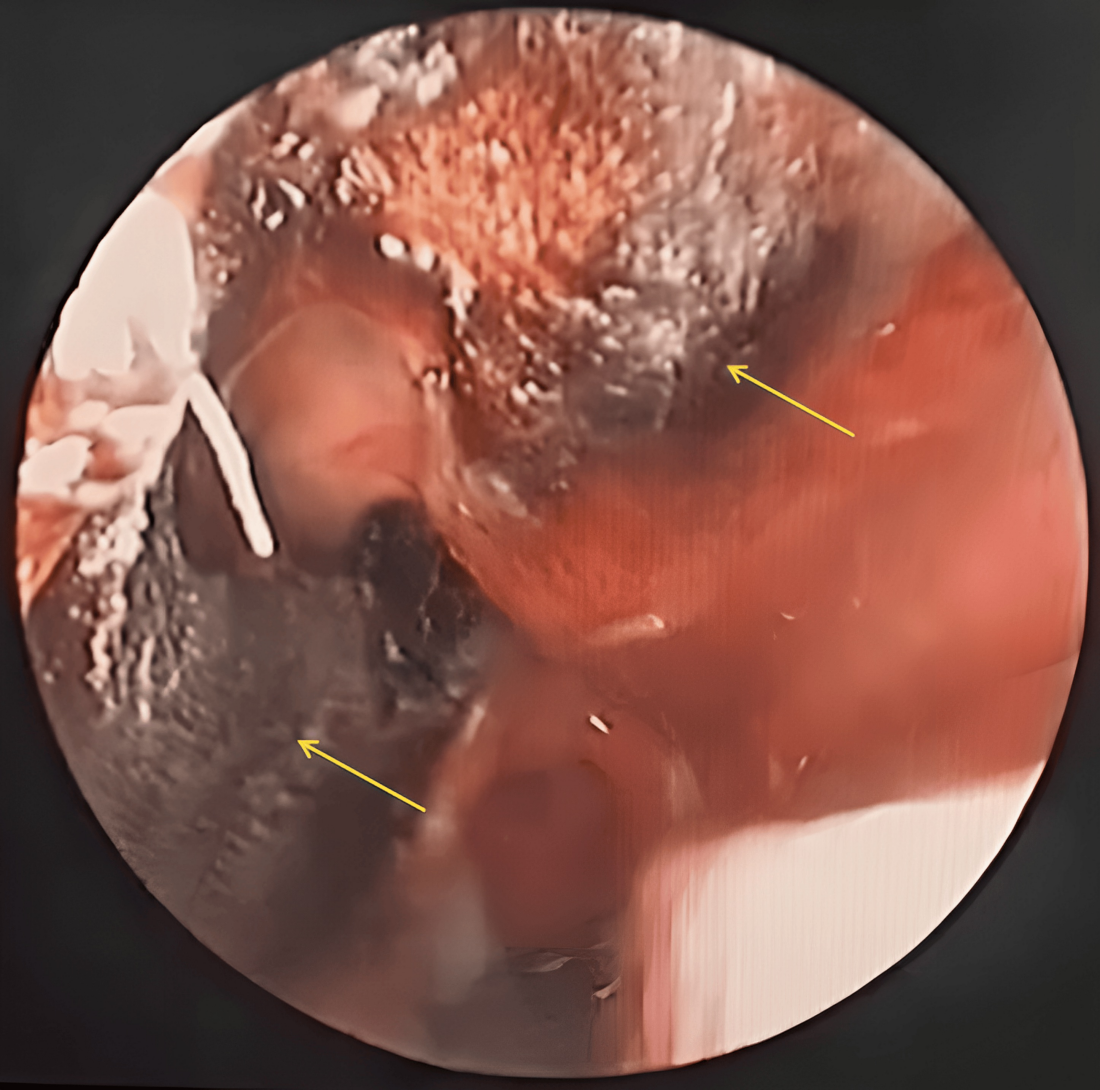
Rigid nasal endoscopy showing blackish discoloration and the crustation of the nasal septum and the lateral nasal wall (arrows).

After 10 days of her admission, the patient was referred to the otolaryngology department for her complaint, that is, persistent facial pain/pressure on the left side and nasal dryness. There was no history of nasal obstruction, discharge, anosmia, headache, diplopia, or decreased vision. During the consultation, the patient was conscious, alert, afebrile, and vitally stable. However, she looked ill, fatigued, and dehydrated. Ocular examination showed that there was normal extraocular movement, no orbital or facial asymmetry, and no decreased facial sensation. In addition, the ear, nose, and throat examination showed hard wax in the right ear, otitis media with effusion in the left ear, and dry blackish secretions adhering to the septum and the middle turbinate of the nose. The rigid nasal endoscopy showed blackish discoloration and the crustation of the nasal septum (around 2 cm), the middle turbinate, and the lateral nasal wall. In addition, there was a nasopharyngeal asymmetry (Figure 1).

**Figure 2:**
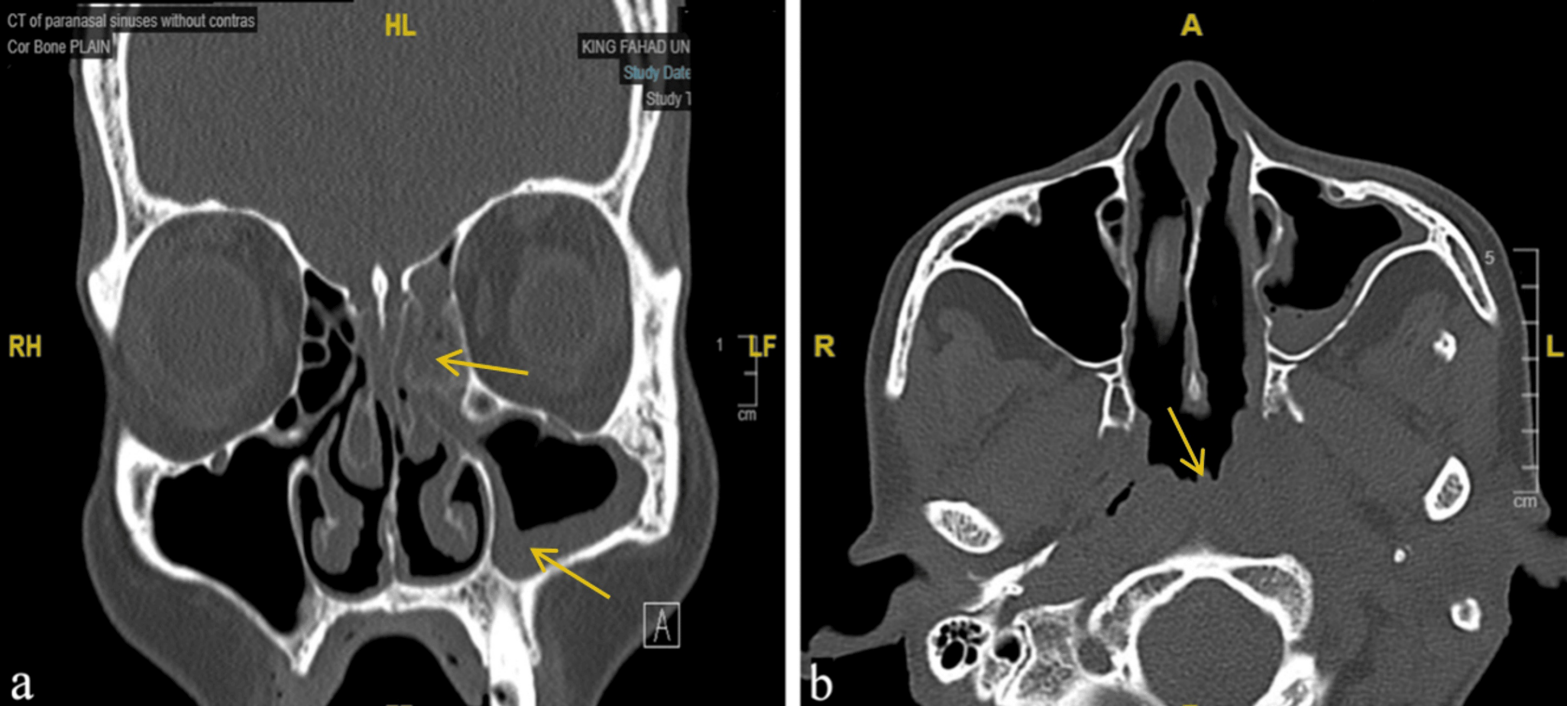
(a) A coronal view of the sinuses showing the complete opacification of the anterior and posterior ethmoid and mucosal thickening of the left maxillary sinus (arrows). (b) An axial view of the sinuses demonstrating the nasopharyngeal asymmetry (arrow).

**Figure 3:**
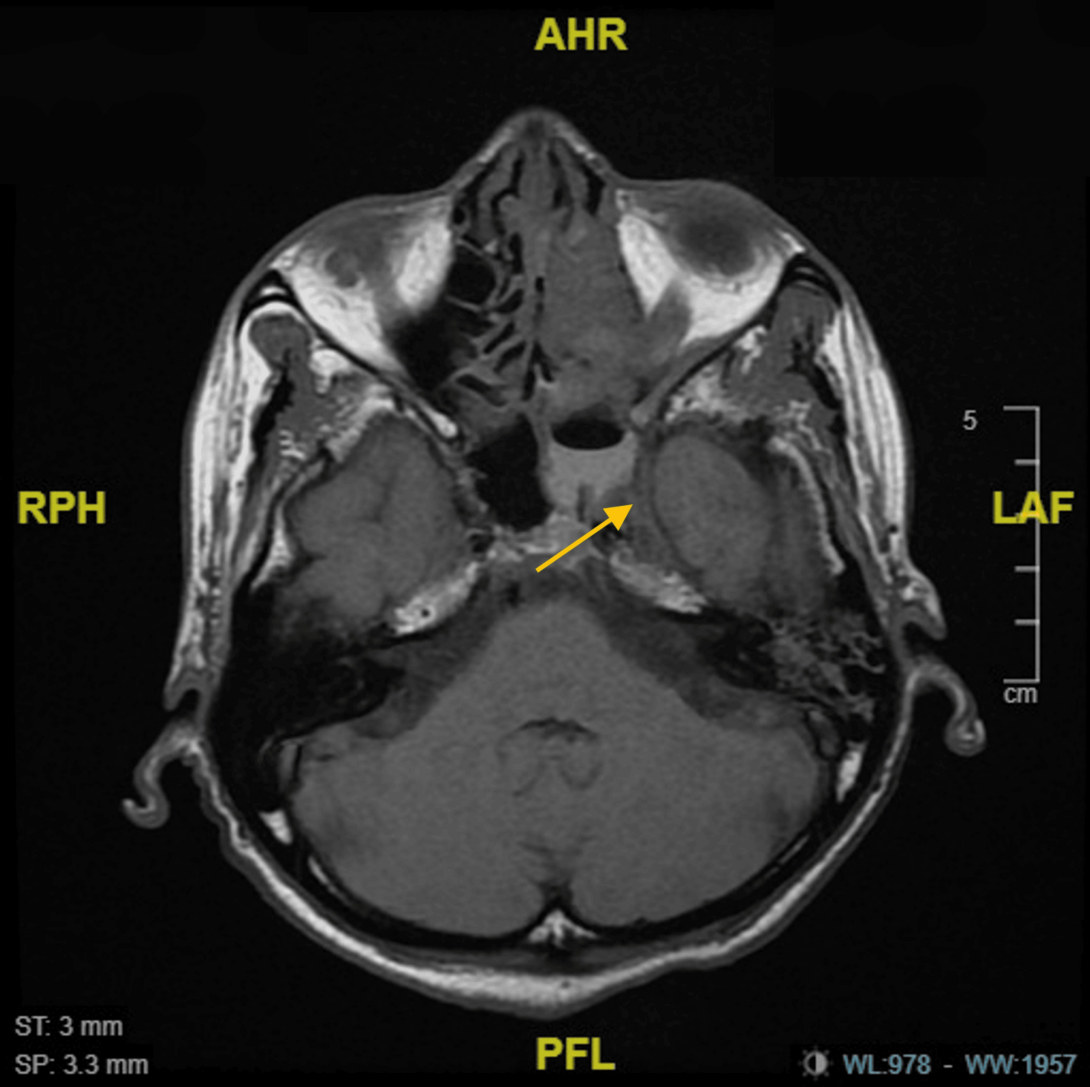
An axial (T1) MRI view showing the intracranial extension at the level of the fovea ethmoidalis (arrow).

A sinus computerized tomography (CT) scan without contrast was done and it showed a complete opacification of the anterior and posterior ethmoid, mucosal thickening of the left maxillary sinus, and a nasopharyngeal asymmetry (Figures 2a, 2b). Furthermore, a magnetic resonance imaging (MRI) was performed to rule out possible ocular and intracranial extension. The results showed an intracranial extension at the level of the fovea ethmoidalis, as seen in Figure 3.

After 11 days of her admission, the further endoscopic nasal examination was conducted in the operating room under general anesthesia and a biopsy was taken from the nasal septum, the middle turbinate, the lateral nasal wall, and the nasopharynx and was sent for fungal culture. The fungal culture results revealed an isolated *R. arrhizus* incidence, which confirmed the diagnosis of mucormycosis. Consequently, the patient was scheduled for functional endoscopic sinus surgery (FESS) and was started on lipid complex amphotericin B at a dose of 175mg daily. After 20 days of her admission, FESS was done for the debridement of the eschar, which was found and removed from the nasopharynx, the middle turbinate, and the lateral nasal wall.

**Figure 4:**
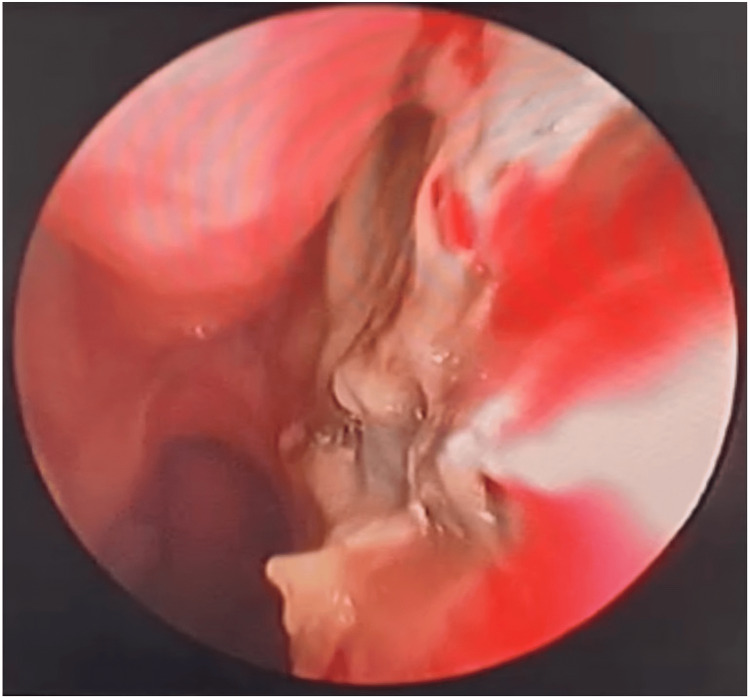
Rigid nasal endoscopy after applying the local miconazole cream.

After an 11-day period of using amphotericin, she developed acute kidney injury (AKI) secondary to amphotericin use which was held for a period of 14 days and switched to clindamycin at a dose of 600mg every six hours. After controlling the AKI, amphotericin was restarted with daily monitoring of the renal function test (RFT), and meropenem at a dose of 500mg twice daily was added with a continuous crust removal every other day, although the patient did not improve in this regimen after 22 days. Hence, local miconazole cream twice per day was added, which showed a significant improvement after application for five weeks (Figure 4). The patient finally completed a course of two months of intravenous amphotericin then she was discharged with a total hospitalization period of 95 days after ensuring resolved AKI, normal inflammatory markers, complete crust removal, and clear nasal cavity with no eschar. The patient was given a follow-up visit to the clinic three months after her discharge and showed no recurrence of symptoms.

## Discussion

During the coronavirus disease (COVID-19) pandemic, the incidence of mucormycosis increased as a secondary infection of COVID-19. It is a rare form of infection, but it is considered one of the most serious infections affecting immunocompromised individuals. Nevertheless, few studies have reported the occurrence of mucormycosis in an immunocompetent healthy individual [10]. The most common risk factor of this condition is DM, with a 36% incidence, followed by hematological malignancy (17%) and solid organ transplant (12%) [11]. The incidence of mucormycosis increases with uncontrolled diabetes as those patients are prone to developing DKA, which increases the serum iron levels in the body and, in turn, supports the growth of fungal pathogens [12]. However, other risk factors, such as prolonged neutropenia, corticosteroid therapy, voriconazole prophylaxis, severe burns, trauma, acquired immunodeficiency syndrome, intravenous drug abuse, and malnutrition, have been identified [13].

Few comprehensive studies have been conducted to analyze the exact pathogen that is linked to mucormycosis in the population. However, several pathogens of the *Mucorales* order have been linked to this disease, such as, *Mucor*, *Rhizomucor*, *Cunninghamella*, *Absidia*, and *R. oryzae* as the most common pathogen with a 60% incidence [4-6]. Similarly, the fungal culture of our case resulted in an incidence of *R. arrhizus*.

Mucormycosis is a rapidly progressing infection with a variety of clinical presentations, which makes early diagnosis difficult. The early presentation depends on the type of the disease, which can be divided into five types, rhino-orbito-cerebral mucormycosis, pulmonary mucormycosis, cutaneous mucormycosis, disseminated mucormycosis, and gastrointestinal mucormycosis. Rhino-orbito-cerebral mucormycosis is the most common type, which affects the nasal and oral cavity, the sinuses, and the brain as seen in this case [14]. Common symptoms of this type are facial pain, proptosis, amaurosis, and palatal/nasal ulcers with central necrosis classically seen as a dark patch [15]. Although our patient presented with a sore throat and generalized fatigue at the beginning, we observed a persistent pain/pressure on the left side of the face, nasal dryness, and blackish discoloration in the nose after monitoring her condition daily.

The overall survival rate for mucormycosis with cerebral involvement is 15%, while it is nearly 50% for patients with rhino-orbital infection [15,16]. Hence, the diagnosis of this condition should not only be based on the clinical presentation, but also on the culture and radiological investigations. CT scan and MRI are considered the mainstay for early diagnosis of mucormycosis, even before the clinical symptoms present, making them instrumental in preventing the complications of this condition [17].

The treatment of mucormycosis consists of three steps: treating the underlying disease, medical management with an antifungal agent, and surgical debridement. Previous studies reported the use of either just medical management or just surgical debridement; however, an antifungal agent in combination with surgical debridement was the most effective reported method for treating mucormycosis. Amphotericin B (5-10mg/kg/day) was the most common antifungal agent used with an average reported duration of 72 days [18]. Additionally, posaconazole, caspofungin, and anidulafungin were used in combination with amphotericin B in previous studies, with variable results [15]. Moreover, adjunctive modalities include hyperbaric oxygen therapy and local treatment with amphotericin B [19]. Hyperbaric oxygen therapy helps through increasing the oxygenation of the affected tissues distal to the occluded vessel and decreasing local acidosis and enhancing the activity of the fungicidal medication [20]. After controlling the DKA, our patient was treated with a combination of amphotericin B, miconazole, and surgical debridement. Additionally, local miconazole cream was added later as an adjunctive therapy for a period of five weeks. To the best of our knowledge, this is the first study that has reported the use of local miconazole cream in treating sinus mucormycosis, showing a highly effective result. Unlike the previous studies, this study reported a case of mucormycosis with a good prognosis, which was due to the early diagnosis and management and the use of local miconazole cream as an adjunctive therapy.

## Conclusions

Mucormycosis is a rare and life-threatening invasive fungal infection, which needs to be suspected in any diabetic or immunocompromised patient. Although the prognosis of this condition is poor, early diagnosis and treatment can improve the prognosis and are vital to preventing fatality. Our patient was treated with a combination of surgery and medical therapy for 95 days after improvement. In this study, local miconazole cream was used as an adjunctive therapy for the first time in the literature. Thus, this highlights the necessity for extensive future clinical trials to validate its effectiveness.
